# Partnership and Motivations for Starting a Family of One’s Own in the Opinions of Students with Disabilities

**DOI:** 10.3390/ijerph20115971

**Published:** 2023-05-27

**Authors:** Maria Łukaszek, Małgorzata Zaborniak-Sobczak, Remigiusz Kijak

**Affiliations:** 1 Department of Resocialization Pedagogy, Faculty of Pedagogy, University of Rzeszów, 35-010 Rzeszów, Poland; 2 Department of Disability Research, University of Rzeszów, 35-010 Rzeszów, Poland; mzaborniak@ur.edu.pl (M.Z.-S.); r.kijak@uw.edu.pl (R.K.); 3 Psychological and Pedagogical Counselling Centre No 1 in Rzeszów, 35-005 Rzeszów, Poland; 4 Department of Biomedical Foundations of Development and Sexology, University of Warsaw, 00-927 Warsaw, Poland

**Keywords:** students, disability, own family, intimate relationships, partnership, mental health

## Abstract

Creating and maintaining stable, happy intimate relationships is a right every individual has. Previous research has shown that people with disabilities are at risk of building unsatisfactory partner relationships. The aim of the study was to ascertain the beliefs of students with disabilities concerning their motives for starting families and, in regard to potential partners, their tolerance for risky life experiences and the personal qualities accepted. A cross-sectional study was conducted with a sample of 2847 university students in southeastern Poland. It was found that the following motives for entering into a permanent relationship were considered more important by students with disabilities than they were by students without disabilities: enhancement of self-esteem (*p* = 0.001), high economic potential of a partner (*p* = 0.007) and a shared system of values and interests (*p* = 0.036). Love (*p* = 0.031) and the mental qualities (*p* = 0.010) of a partner were considered less important by students with disabilities than they were by students without disabilities. Moreover, students with disabilities are far more likely than students without disabilities to accept disability (*p* < 0.001) in potential partners. They are also significantly more willing to enter into relationships with people who have risky life experiences, even in the form of violence against previous life partners (*p* < 0.015) and children (*p* = 0.001), addiction to alcohol (*p* < 0.001) or drugs (*p* = 0.01) and the resulting treatment, and those having served time in prison (*p* = 0.034). Educational and institutional support for students with disabilities should be intensified with regard to partner selection being satisfactory to both partners.

## 1. Introduction

Traditionally accomplishing the developmental tasks of early adulthood allows the person to assimilate three main social roles: worker, spouse/life partner and parent [1,2,3]. Attempting to create and maintain permanent intimate relationships includes both people with and without disabilities, and the right to sexuality and relationships are rights of every human being. In the United Nations Convention on the Rights of Persons with Disabilities [4], it is emphasized/legislated that people with disabilities have the right to independence and social inclusion (Article 19), respect for privacy (Article 22) and non-discrimination in matters relating to marriage, family, parenthood and intimate relationships (Article 23). A satisfying family life and high quality, happy partner relationships can support the mental health and well-being of this group of people [5,6,7], as well as enhance self-esteem and lower stress levels [8]. There is evidence that relationships with one’s immediate social environment (e.g., family, friends, and co-workers) have a beneficial effect on mental health, while social isolation or lack of close social ties is associated with poorer health and increased risk of mortality. These relationships affect the general population, but are particularly relevant to people with disabilities, due to their limited participation in society. Disability is linked to the problem of public health. People with disability tend to have fewer opportunities to participate in society. Decreased mental health is a major health burden worldwide, and particularly in populations with disabilities [7].

### 1.1. People with Disabilities in Polish Society

People with disabilities are a part of any society. It is now accepted that disability is an evolving concept, and denotes the interaction between people with dysfunctions and barriers due to human and environmental attitudes that hinder these people from participating fully and effectively in society, on an equal basis with others [9]. For statistical purposes, disability is studied based on two criteria: 1. legal (formal)—legal disability, and 2. subjective (self-assessment)—biological disability. Thus, it is assumed that a person with a disability in legal terms is a person who has a relevant certificate issued by an authorized body. Our own research also adopted this way of selecting people with disability from the general sample of respondents (that is, on the basis of holding a disability certificate). The system of disability evaluation in Poland is not uniform. It is defined by two legal acts:

1. Act of 27 August 1997 on Vocational and Social Rehabilitation and Employment of Persons with Disabilities [10], which, in Article 2, defines disability as a permanent or temporary inability to fulfill social roles due to permanent or long-term impairment of bodily functions resulting, in particular, in inability to work. In Article 3, degrees of disability for certification are established: considerable, moderate, and slight. Article 4a mentions that certification for children up to sixteen is provided without any decision on the level of disability. Disability evaluation boards are county (first instance) and provincial (second instance). The final instance concerns district labor and social security courts;
2. Act of 17 December 1998 on Old-Age and Disability Pensions from the Social Insurance Fund [11], which, in article 12, defines a person who is incapable of working as one who has completely or partially lost the ability to work for gain due to impairment of bodily functions and does not show promise to regain the ability to work after retraining. Evaluations regarding inability to work, inability to lead an independent life, and the advisability of retraining, issued by the Social Security Administration, are subject to translation into disability certificates in accordance with Article 5 of the aforementioned Act of 27 August 1997 on Vocational and Social Rehabilitation and Employment of Persons with Disabilities.

For a person to be considered disabled in the legal--formal sense, he or she must have one of the above-described certificates [12].

In the 2020/2021 academic year, 19,600 people with disabilities were studying at universities (1.6% of the total number of students), and 5200 graduated (1.8% of the total number of graduates). Among PhD students, 3.3% were people with disabilities (800 people) [12]. 

Preparing people with disabilities for their roles as life partners/husbands or wives is sometimes a taboo subject, and those who attempt to break it are branded within society. It seems that, despite the many declarations made, neither teachers and parents nor society as a whole give this group the right to form any kind of intimate relationship. Their emotional needs, which could be fulfilled through creating bonds and a sense of belonging in a relationship, are ignored, while their sexual needs are sabotaged. For example, as many as 26% of Poles categorically oppose marriage of people with intellectual disabilities [13]. Social research conducted among people with disabilities has, however, shown that people with intellectual disabilities are able to pursue successful intimate relationships [14] and marriages of people with disabilities are characterized by significantly higher stability and cohesion compared to marriages of people without disabilities, as well as increased satisfaction over time [15]. These findings, however, only minimally influence public opinion.

### 1.2. Intimate Relationships of People with Disabilities in Research to Date

The prerequisite for building a permanent, mutually satisfying intimate relationship is having a mature motivation for entering the relationship and a conscious, thoughtful choice of a committed and supportive life partner. This demand takes on particular importance for people with disabilities. Available research shows the following:

- Among adolescents with disabilities, experiences with partner relationships and sexual activity occur less frequently and later in life (this is partly differentiated by gender and type of disability). Reasons for this, include visibility of the disability and the resulting smaller range of potential partners, lack of self-confidence, low self-esteem (this is more common among girls with physical disabilities than boys with disabilities), difficulty accepting physicality, overprotective parents, stigma, and negative social attitudes [16]. It was found, for example, that the majority of young adults (80%) from Australia and Hong Kong perceive a potential partner having a disability as an obstacle to dating [17];
- People growing up with a disability are less likely to marry, compared to people without disability [18,19,20], but the trend toward less frequent marriage also applies to people without disabilities today, as other forms of relationship life (for example, partnerships) are available. In the case of women, a high level of physical disability reduces the likelihood of marriage by 22% [21];
- People with disabilities are more likely than others to enter into partnerships with partners with disabilities [16,22],
- People with disabilities who wish to marry or form a permanent intimate relationship face a number of problems, such as the following: limited opportunities to get to know a partner, manifestations of discrimination by potential partners, and negative evaluation of their own bodies and sexuality [21,23,24]. In addition, their willingness and attempts to form permanent intimate relationships may be criticized by their partners, as well as by their families, and even by others within the social environment [23];
- People with disabilities are more likely to remain single for long periods of time, although there is less evidence of differences in relationship breakup rates for those who enter said relationships [19];
- Of people with disabilities living in permanent relationships 28% declared themselves to be dissatisfied with said relationships [8], while, in comparison, only 5% in a nationwide sample of Poles aged 18–49 in intimate relationships declared themselves dissatisfied [25];
- People with disabilities who are in committed relationships are more likely to be victims [26,27,28,29,30], and up to twice as much so as their peers without disabilities [31]. These victims with disabilities are primarily women [27,29,32,33,34,35]. These women suffer various forms of partner violence from 40% to 100% more often than women without disabilities [30,33,35]. Furthermore, their aggressors do not feel remorse, nor do they try to make up for the suffering inflicted, as tend to be the case when aggressors inflict suffering on partners without disabilities [33]. No repentance or honeymoon phases, as referred to in the Walker Cycle of Violence, is observed here [36]. In addition, women with disabilities are convinced by the social environment that they should accept violence out of gratitude that their partner tolerates their disabilities [33];
- People with disabilities, become victims of sexual violence by their intimate partners far more often than non-disabled people [37] It was also found that poorer health of the victim was associated with higher rates of experiencing this form of violence [38];
- Victims of violence who have disabilities bear higher costs from traumatic experiences and were more likely to report symptoms of depression, self-harm and stress [39], as well as being more likely to be victimized [28]. These facts are primarily associated with the social isolation and discrimination felt by victims with disabilities [40].

In the context of these considerations, it is important to note that studies have found that university attendance and labor force participation among people with disabilities are correlated with higher rates of marriage [22].

The subjects of our research were university students who were People With Disability (PWD) and university students who were People Without Disability (PWOD). Our subjects were members of a group of young adults for whom finding an intimate partner is one of their most important developmental tasks. Successful accomplishment of this task manifests itself primarily in the ability to create permanent and satisfying relationships for both partners. Young adults with disabilities living in Poland often fail when trying to build stable intimate relationships. For example, among adult Poles with physical disabilities, only 54% are in a marriage and 4% in a permanent partner relationship [41].

The subject of our own research was to investigate students’ motives for starting a family, and their beliefs about accepted personal qualities and the risky life experiences of potential life partners. The primary goal of the current analysis was to assess whether the disabilities of the students surveyed differentiated these opinions. The study attempted to answer the following research questions:

1. Is there a difference, and, if so, what is the difference, in motives for starting a family declared by students with and without disabilities?
2. Is there a difference, and, if so, what is the difference, in the qualities of potential life partners acceptable to students with and without disabilities?
3. Is there a difference, and, if so, what is the difference, in the level of tolerance of risky life experiences of potential life partners declared by students with and without disabilities?

## 2. Materials and Methods

### 2.1. Study Design and Sample

The material was based on part of the results collected during the 2020/2021 project, “Preferred and accepted type of life partner and family life by students with and without disability”.

Based on the statistical yearbook, the size of the general population of students in the three provinces of the southeastern region of Poland (Podkarpackie, Małopolskie, Lubelskie) was estimated, which amounted to 256,438 people, of which women accounted for 58.6% and men 41.4%. People with disabilities accounted for 1.66%. As we wanted a statistical error of less than 2%, the minimum sample size was estimated at 2500 people [42]. The research sample finally consisted of 2847 randomly selected students, taking into account criteria of field of science studied and gender, studying at universities and colleges in three provinces (Podkarpackie, Małopolskie, Lubelskie).

Women constituted 75.7% of the study sample (76.5% of the women were in the people without disability group and 63.5% in the people with disability group). Men constituted 24.3% of the study sample (23.5% of the men were in the people without disability group and 36.5% in the people with disability group). Of the total number of respondents, 6% were people with disabilities. Among them, 40% declared slight, 46.5% moderate degree, and 13.5% considerable degrees of disability. 

In the group of students with disabilities, the most frequently declared disabilities were musculoskeletal impairment (22.4%), visual impairment (13.5%), neurological diseases (12.9%), endocrine disorders, metabolic disorders, enzyme disorders, infectious and zoonotic diseases, disfigurement, diseases of the hematopoietic system (11.8%); mental illness (10%); voice, speech and hearing disorders (8.2%), and respiratory diseases (4.7%) and mental retardation (4.2%). The remaining disabilities involved a percentage of less than 4% (it should be noted that some students had certificates indicating several disabilities). Of the total number of people with disabilities surveyed, 15.9% said they required constant assistance from others.

Considering the field of study, 38.7% of people were students of social sciences, 15.3% of medical sciences and health sciences, 13.9% of humanities, 13.4% of engineering and technical sciences, 10.3% of sciences and natural sciences, 4.1% of agricultural sciences, 2.4% of arts, and 0.5% of theological sciences (1.4% did not answer this question). Among the respondents, 49.8% said they were in a permanent marriage or partnership (49.9% among people without disabilities, 47.1% among people with disabilities). 

### 2.2. Survey Measures, Procedure

The study used a diagnostic survey. The tool was a survey questionnaire by Maria Łukaszek, consisting of 38 questions or blocks of questions. The questions and scales included in the questionnaire were based on numerous theoretical models of relationship functioning included in the literature (e.g., M. Plopy [43]), some of which included standardized diagnostic tools. The whole questionnaire was analyzed by competent judges. Pilot testing of the tool and the first survey of a sample of adult high school students (*n* = 1150) was conducted by the tool’s author in 2020. 

In the material presented here, results from 3 measurement scales relating to dependent variables (Appendix A Table A1, Table A2 and Table A3) were analyzed. The independent variable was determined by the question about having a disability degree certificate.

In the validation process, the measurement scales used were found to be reliable. The Cronbach’s Alpha internal consistency coefficient measurement index of all scales ranged from 0.86 to 0.95.

The first of the scales, “Scale of motives for starting a family” (to marry or form a permanent partnership) (Cronbach’s Alpha coefficient—0.857) consisted of 22 statements (specific measures) (Appendix A Table A1) relating to eleven categories of motives (synthetic measures):

- physical qualities of the partner,
- mental qualities of the partner,
- emotional commitment of partner,
- high economic potential of the partner,
- striving to build a community,
- feeling better about oneself,
- social pressure,
- sexual benefits,
- a common system of values and interests,
- a sense of internal pressure to form a relationship,
- pregnancy and the fear of its consequences.

Respondents were asked to rate the importance of 22 motives that would make them choose to start a family, marry, or have a permanent partner relationship. A four-point scale was used for evaluation: “motive is very important”, “important”, “rather unimportant”, “unimportant”.

The second scale was the “Scale of acceptance of qualities of potential life partners” (Cronbach’s Alpha coefficient—0.892) and it consisted of 31 sentences (specific measures) (Appendix A Table A2) relating to seven categories of qualities (synthetic measures):

- disability,
- external appearance and care for said appearance,
- sexual scripts,
- lifestyle,
- social status,
- age,
- style of functioning in interpersonal relations.

Respondents were asked to rate the extent to which they would accept each of the 31 qualities listed in their potential life partner. Respondents made their choices on a five-point scale: “I strongly accept this feature”, “I rather accept”, “it is difficult to say”, “I rather do not accept”, and “I definitely do not accept”.

The third of the scales, “Scale of tolerance level for risky experiences of potential life partners” (Appendix A Table A3) (Cronbach’s Alpha coefficient—0.948), consisted of 30 sentences (specific measures) depicting a variety of broadly defined risky experiences. Risky experiences were considered to be those whose consequences may significantly impede the formation of lasting, functional intimate relationships in the future and interfere with the successful performance of marital or partnership roles. These experiences are associated with the occurrence of negative consequences for health (physical, mental and sexual) and a significant reduction in social status (including legal standing). The list of risky experiences was reviewed by two separate teams of expert judges and during a pilot study. The respondents’ task was to answer the question “Would you choose to create a family (cohabitation or marriage) with a person who had the following experiences”, considering the 30 listed experiences. Responses were given on a five-point scale: “definitely yes”, “rather yes”, “hard to say”, “rather no”, and “definitely no”. The 30 sentences describing the various risky experiences of potential life partners were combined into five risk areas:

- experiences related to addiction to psychoactive substances or behavioral addictions,
- risky sexual experiences,
- experiences related to breaking legal norms/legal punishment,
- experiences with mental health disorders,
- being utterly unfit for the role of parent.

The independent variable “having a disability” was determined based on a question about the degree of disability specified in the disability certificate (possible answers were: “I do not have a disability certificate”, “considerable”, “moderate”, “slight”).

The implementation of the study took place in an online format, by means of a Computer Assisted Web Interview. The survey questionnaire was created using Google Forms. The authors requested that the authorities of the higher education institutions directed students of the selected faculties, representing particular fields of science, to fill out questionnaires placed in the Internet cloud. Students were invited to participate in the study via institutional e-mail, educational platforms of individual universities in southeastern Poland, and the websites of the student self-governments of the designated universities, as well as through the Disability Affairs Offices operating in the universities. The online survey questionnaire was sent to students in the form of a link. The decision was made to use an electronic questionnaire because it increased convenience and anonymity and resulted in lower costs, while avoiding wasting of paper. It also resulted in the immediate storage of all responses in the same database and a higher response rate.

### 2.3. Ethics of the Study

The procedures of the implemented project fully respected the recommendations included in the Declaration of Helsinki. Respondents were guaranteed informed, anonymous, and voluntary participation in the survey. During the survey, respondents were also not required to answer all questions. The design of the sheet allowed them to bypass questions they did not want to answer, such as for personal reasons. 

### 2.4. Analytical Approach

The first stage analyzed the motives for starting a family as declared by university students with and without disabilities. The “Scale of motives for starting a family” (getting married or creating a permanent partnership) used in the survey was a 4-point scale, where arithmetic averages were calculated based on the points for each answer: motive rated as very important—3 points, important—2 points, rather unimportant—1 point, unimportant—0 points. Measures of the intensity of beliefs about the importance of each motive for starting a family were constructed, taking values from 0 to 3 points. Analyses were conducted at the detailed level (comparing 22 motives—specific measures) and at the synthetic level (referring to 11 categories of motives—synthetic measures). To determine the incidence and statistical significance of differences between the means in the analyzed groups, analyses were carried out with a t-test for heterogeneous variances. This was the Cochran–Cox test.

The second stage of analysis compared differences in the level of acceptance of the qualities of potential life partners declared by students functioning with and without disabilities. The results obtained from the measurement of the “Scale of acceptance of qualities of potential life partners” were taken into account. This was a 5-point scale, where arithmetic averages were calculated based on the points awarded for each response: strongly accept this quality—4 points, rather accept—3 points, hard to say—2 points, rather do not accept—1 point, strongly do not accept—0 points. Measures of the intensity of acceptance of the characteristics of potential life partners were constructed, taking values from 0 to 4 points. Analyses were conducted at the detailed level (considering 31 qualities of potential life partners—specific measures) and at the synthetic level (referring to 7 categories of qualities—synthetic measures). Analyses were carried out with a t-test for heterogeneous variances (Cochran–Cox test) to identify and assess the statistical significance of differences between mean scores in the analyzed populations. 

The third stage of the empirical study compared differences in the level of tolerance for risky experiences of potential life partners declared by students with and without disabilities. The results from the measurement of the “Scale of tolerance of risky life experiences of potential life partners” were used. This was a 5-point scale, where arithmetic averages were calculated based on the points awarded for each answer: definitely yes—4 points, rather yes—3 points, hard to say—2 points, rather no—1 point, definitely no—0 points. Measures of the tolerance level of risky experiences of potential life partners were constructed, taking values from 0 to 4 points. To determine the existence and statistical significance of differences between the means of measures (for 30 sentences—specific measures) in the analyzed populations, analyses were carried out with a t-test for heterogeneous variances (Cochran–Cox test). In addition, 5 categories of risky problem experiences were identified: 1. addictions, 2. sexual functioning, 3. criminal record/legal punishment, 4. mental health, and 5. realization of role of parent (Appendix A Table A3). Those of the 30 risky experiences that met the substantive criteria were qualified for each category. Therefore, some of the sentences fitted two categories, which meant the categories were inseparable. Then, within each of the five categories (synthetic measures), an analysis was conducted of the differences (Cochran–Cox *t*-test) between the tolerance levels of people with and without disabilities.

The analyses were performed with the use of the specialized STATISTICA 12 statistical package (StatSoft, Cracow, Poland).

## 3. Results

During the study, an attempt was made to find out whether there was a difference, and, if so, what the difference was regarding the motives for starting a family as declared by students with and without disabilities (Table 1).

The survey showed that students in the PWD group, like those in the PWOD group, declared that when deciding to create a marriage or permanent partnership, they would be guided primarily by the following: “a feeling of love for their partner”, “feeling that they are loved by their partner”, “the partner’s personality appeals to me”, “a desire to form a permanent relationship”, and “a similar life value system”. For both groups of students surveyed, “the mental balance of a potential partner” and “the fact of a long-term acquaintance/friendship” were also important. 

However, detailed analyses of variance indicated statistically significant differences in how the two groups of respondents ranked each motive. It was significant that love, despite ranking high in the hierarchy of motives for starting a family, was a weaker justification for building a relationship for students with disabilities than for people without disabilities. It was found that both “feeling of love for their partner” (averages: PWD = 2.80, PWOD = 2.89; *p* = 0.022) and “feeling of being loved by the partner” (averages: PWD = 2.78 and PWOD = 2.87; *p* = 0.041) were ranked lower by people with disabilities. “Partner’s mental balance” was also more important for students without disabilities (PWOD = 2.54) than for those with disabilities (PWD = 2.33; *p* = 0.001). 

The choice of motives that ranked highest in the hierarchy was not statistically significantly different in the two groups of students surveyed.

In addition, a significantly higher average score was found among people with disabilities in categories related to confronting public opinion. Students with disabilities considered “avoiding ‘badmouthing’ by people due to pregnancy” (PWD = 0.89, PWOD = 0.58; *p* < 0.001) to be a much more important motive for creating a permanent relationship. Students with disabilities would be willing to start a family under the influence of “persuasion by family or friends” at a higher rate (PWD = 0.71) than students without disabilities (PWOD = 0.51; *p* = 0.004).

Students with disabilities also gave significantly higher ranking to the motive of “proving to others that I can get an attractive woman/man on a permanent basis” (PWD = 1.04, PWOD = 0.73; *p* < 0.001). Similarly, they ranked “proving to myself that I am attractive enough for that kind of man/woman to choose me as a partner” (PWD = 1.3, PWOD = 1.08; *p* = 0.005) higher than non-disabled students.

It is noteworthy that students with disabilities, far more than non-disabled students, were guided in their decisions to build permanent relationships by the prospect of the benefits of their partner’s high material status and excellent physical fitness. Disabled people considered the following as a significantly more important motive for building a partner relationship than people without disability (PWD = 1.08 and PWOD = 0.85; *p* = 0.002)—“possession of extensive material resources by the partner, for example: apartment, high-end car, money”. They also gave a higher ranking (PWD = 1.45 and PWOD = 1.27; *p* = 0.016) to “partner’s capabilities that guarantee the achievement of high material status in the future (e.g., high education, steady, well-paid work, the chance to inherit property from relatives)”.

In exploring the results, 22 motives for starting a family (Table 1) were combined into 11 categories (Table 2) creating synthetic measures, and then the averages of the created measures in each category were subjected to analysis. The table shows that the most important categories of motives for marriage or starting a partner relationship for all the students surveyed were: “emotional commitment” i.e., mutual love between partners, “psychological qualities of a potential partner” and “striving to build a community”.

However, numerous statistically significant differences were found between the categories of motives indicated by the groups of students with and without disabilities. It should be noted that, for students with disabilities, by far the more important categories of reasons for starting a family were “feeling better about oneself” (PWD = 1.17, PWOD = 0.9; *p* = 0.001), “having a partner with high economic potential” (PWD = 1.26, PWOD = 1.06; *p* = 0.007) and “pregnancy” (PWD = 1.1, PWOD = 0.91; *p* = 0.01). Students with disabilities also put a slightly higher value on “shared value system and similar interests of partners”, (*p* = 0.036) “physical qualities of the partner”, (*p* = 0.085) and “peer pressure for them to enter into a relationship” (*p* = 0.145). “Commitment, or mutual love” of partners for students with disabilities, despite its high ranking in the hierarchy of motives for starting a family, was of much lower importance than for their peers without disabilities (PWD = 2.79, PWOD—2.88; *p* = 0.031). 

The study sought to find out if there was a difference, and, if so, what the difference was, in the characteristics of potential life partners accepted by students with and without disabilities.

During the diagnostic survey, respondents were asked to indicate on a five-point scale how much they approved of the listed 31 qualities in a potential life partner (specific measures). During the analysis, these qualities were grouped into 7 categories (synthetic measures) (Table 3). 

The least accepted qualities by students with disabilities and students without disabilities concerned disability of potential life partners and sexual scripts (patterns).

However, an analysis found that the ranks given by students with and without disabilities differed in a statistically significant way, primarily in specific measures relating to acceptance of a potential partner’s disability, appearance, age and social status. 

Respondents with disabilities were significantly more accepting of “physical disability” (PWD = 2.50, PWOD = 2.07; *p* < 0.001), “hearing impairment” (PWD = 2.80 and PWOD = 2.58; *p* = 0.004) and “mild intellectual disability” (PWD = 2.16, PWOD = 1.91, *p* = 0.005) in potential life partners than respondents without disabilities. In contrast, they gave significantly lower ranks than students without disabilities to qualities related to the attractive physical appearance of their partners, although there were no statistically significant differences here. Students with disabilities placed a lower value on both “physical attractiveness” of potential partners (PWD = 3.35, PWOD = 3.48; *p* = 0.083), as well as “partners’ care for their own physical appearance” (PWD = 3.39, PWOD = 3.55; *p* = 0.007) and “focus on physical fitness” (PWD = 3.53, PWOD = 3.61; *p* = 0.110). In contrast, they accepted their partner being “overweight” to a higher degree than students without disabilities (PWD = 2.45, PWOD = 2.36; *p* = 0.281).

It was found that students with disabilities perceived the social status of potential life partners differently to students without disabilities. They were far less accepting of “partner’s education higher than their own” (PWD = 3.31, PWOD = 3.48; *p* = 0.018) and “partner’s job at a higher position than they hold themselves” (PWD = 3.30, PWOD = 3.42; *p* = 0.061).

The data showed that people with disabilities were also significantly more likely than people without disabilities to accept life partners that were “younger than themselves” (PWD = 3.02, PWOD = 2.83; *p* = 0.030), and, thus, less likely than their peers without disabilities to accept “older partners” (PWD = 3.32, PWOD = 3.47; *p* = 0.045).

Analyzing the accepted lifestyle traits of potential life partners, it was observed that students with disabilities, compared to those without disabilities, gave significantly lower ranks to “a partner’s focus on an active cultural and social life” (PWD = 3.23, PWOD = 3.39; *p* = 0.027), while they preferred “spending leisure time at home and keeping social contacts to a minimum” (PWD = 2.54, PWOD = 2.38; *p* = 0.086). In terms of interpersonal relationships, people with disabilities were less accepting of “thoughtfulness, caring” in their partners (PWD = 3.61, PWOD = 3.76; *p* = 0.018), which could be considered surprising.

Analysis of synthetic measures in seven categories showed statistically significant differences only in the disability category. It was found that students with disabilities would be significantly more likely than students without disabilities to accept disability in a potential life partner (PWD = 3.61, PWOD = 3.36; *p* < 0.001).

One of the questions we sought to have answered during the research project was whether there was a difference, and, if so, what the difference was, in the level of tolerance for risky life experiences of potential life partners declared by students with and without disabilities. 

It was found that the average levels of tolerance of risky life experiences of potential life partners for the entire scale (30 statements—Table 4), shown by students with disabilities, were significantly higher (PWD = 1.38) than the tolerance levels of students without disabilities (PWOD = 1.25). 

Analysis of the collected research material indicated that students, both with and without disabilities, were willing to accept numerous risky life experiences of potential partners, which might disrupt the successful implementation of marital or partner roles and family functioning in the future. Respondents were most tolerant of “sexual experiences with multiple partners” and “having a child or children from previous relationships”, “treatment for mental disorders”, “computer or computer game addiction”, as well as “marijuana use” and “tobacco abuse”. 

Statistically significant differences in the levels of tolerance of risky experiences of potential life partners were found regarding the majority of sentences concerning the sphere of sexual life. It was revealed that students with disabilities were reconciled at a higher rate to the following facts: “getting STIs from potential life partners” (PWD = 1.38, PWOD = 1.15; *p* = 0.016), “partners providing sex work services” (PWD = 0.99, PWOD = 0.78; *p* = 0.026), “using sexual violence against ex-partners” (PWD = 0.73, PWOD = 0.56; *p* = 0.043) and “committing infidelities on ex-partners” (PWD = 1.01, PWOD = 0.83; *p* = 0.034). They were also more tolerant than students without disabilities toward “having a child or children from other relationships by potential life partners” (PWD = 2.38, PWOD = 2.18; *p* = 0.042).

The declarations of students with disabilities were distinguished (in statistically significant ways) from those of students without disabilities by strong liberalism with regard to specific measures identifying the risky experiences of potential partners related to addiction problems: “treatment for alcoholism” (PWD = 1.38, PWOD = 1.02; *p* < 0.001) and “drug addiction” (PWD = 1.23, PWOD = 0.97; *p* = 0.01). High levels of tolerance (although not significantly different statistically) were also observed for “intoxication by potential life partners with cocaine, amphetamines, ecstasy, LSD or heroin” (PWD = 0.93, PWOD = 0.75; *p* = 0.072) and “alcohol abuse and addiction” (PWD = 1.08, PWOD = 0.92, *p* = 0.072). Students with disabilities also condoned “use of designer drugs by potential life partners” at a higher rate than students without disabilities (PWD = 1.26, PWOD = 1.09, *p* = 0.060).

The levels of tolerance towards the risky experiences of potential life partners presented by students with disabilities differed in statistically significant ways from the levels of tolerance declared by students without disabilities in areas of conflict with the law and being utterly unfit for the role of parent. Respondents with disabilities were found to more highly tolerate “the fact that a potential life partner is serving a sentence for violence against a former partner” (PWD = 0.67, PWOD = 0.47; *p* = 0.015) or “serving a sentence for violence against children” (PWD = 0.70, PWOD = 0.41; *p* = 0.001), “forcing unacceptable forms of sexual activity on partners” (PWD = 0.73, PWOD = 0.56; *p* = 0.043) and also cases of “deprivation of freedom” (PWD = 1.13, PWOD = 0.94; *p* = 0.034). Students with disabilities were also inclined to be more tolerant of potential partners “deprived of parental rights” (PWD = 0.91, PWOD = 0.70; *p* = 0.013). 

A detailed analysis found that students with disabilities presented significantly higher levels of tolerance than the other respondents for “mental illnesses for which potential life partners are being treated” (PWD = 1.49 and PWOD = 1.15; *p* = 0.001).

Tolerable risky life experiences of a potential partner were included in a synthetic measure of risky life experiences (Table 5; Appendix A Table A3). Based on this, it was found that students with disabilities showed a more liberal approach to the risky experiences of potential partners in all categories. 

Students with disabilities were particularly tolerant of potential partners’ experiences indicating “mental health disorders” (SWD = 1.88, SWOD = 1.68; *p* = 0.015), “risky sexual experiences” (SWD = 1.57, SWOD = 1.41; *p* = 0.015), and “addictions” (SWD = 1.41, SWOD = 1.29; *p* = 0.099). 

It was found that of all the categories of tolerated risky experiences, the statistical significance of differences between the responses of students with and without disabilities was most evident in the categories of “being utterly unfit for the role of parent” (*p* = 0.011), “tolerated risky sexual experiences” (*p* = 0.015) and “mental health disorders” (*p* = 0.015).

## 4. Discussion

Marriage and partner relationships of people with disabilities are fraught with societal risk, risk of social stigma and risk of violence [8,14]. For this reason, it is crucial that these individuals have mature motives when starting a family and they should select their life partners in a thoughtful and considerate manner. 

The study found that students with disabilities differed from their peers without disabilities in a number of issues regarding motives for marriage and starting partner relationships, the accepted qualities of potential life partners, and the tolerated risky life experiences of partners. 

### 4.1. Motivation for Starting a Family

It was found that in the hierarchy of motives for starting a family, both for students with disabilities and those without disabilities, among the highest ranked motives were the mutual love of the partners, the psychological qualities of the potential partner and the desire to build a community. However, for students with disabilities, all of these reasons were much less important than for students without disabilities. Analysis revealed that, for students with disabilities, by far the more important reasons for starting a family were feeling better about oneself, having a partner with high economic potential, pregnancy and the social stigma associated with it, and a shared system of values and interests.

The state of our knowledge to date indicates that the choice of a life partner is largely made on the basis of homogamy, that is, similarity of socioeconomic statuses. People also seek to enhance their social standing and income and consider the attractiveness of a potential partner in this context as well. Hence, when talking about selecting a partner, we also talk about the rule of hypergamy, that is, the tendency to select a partner of higher or convergent status. However, it should be noted that for women this important element of socioeconomic status concerns financial and material resources, while for men it is beauty and youthfulness [44]. When discussing the issues of partner selection, it is worth referring to the “theory of individual self-interest”, the premise of which is that in interpersonal relations people are guided by the principle of exchange of goods. Based on this concept, partner selection is based on the balance of potential gains and losses when talking about creating a relationship with a partner. The balance sheet approach results in the selection of the best matrimonial investment [14,45]. The research presented here shows that in groups of people with disabilities, these rules are significantly modified. 

### 4.2. Accepted Qualities of Potential Life Partners

The results of the study showed that students with disabilities differ (in a statistically significant way) from students without disabilities in their acceptance of many characteristics and qualities of potential life partners. They were found to be significantly more likely than people without disabilities to accept potential partners having physical disabilities, hearing impairments and mild intellectual disabilities, and who were younger. This confirmed previous findings that people with disabilities are more likely to accept partners who are also disabled [22]. It seems that, due to stereotypical attitudes toward people with disabilities, described as asexual and dependent [13,40], adolescents with disabilities may be inclined to seek out peers with disabilities who can identify with them [23]. The resulting segregation of people with disabilities within peer networks may increase their visibility to potential partners who are also disabled, and thus decrease their visibility for potential partners without disabilities. Thus, people with disabilities may be disproportionately less likely to marry someone without a disability because they are excluded from peer networks formed by people without disabilities, even if they participate in the same social activities [23]. Connecting through stigmas and choosing self-exclusion leads to entering relationships with people with the stigma of disability [46]. Our own research also confirmed that, in the minds of people with disabilities, there is still the last barrier to integration, which is full acceptance of intermarriage (marriages between people with disabilities and people without disabilities) [47].

During the research presented in this article, it was found that students with disabilities were also less likely than students without disabilities (with a statistical significance of differences) to accept partners who care about their physical appearance, are focused towards a rich cultural and social life, have a higher education than them and, are older, caring and nurturing. Presumably, this fact can be explained by internal barriers against associating with a more physically attractive and more socially active person. According to the study, the belief of people with physical disabilities (especially in the case of women [48]) concerning the shortcomings of their own physicality, made it much more difficult to establish an emotional relationship. According to the study, in the case of women with disabilities choice of partner was determined by entering into an intimate relationship with any interested partner. They especially appreciated people who were not primarily interested in the sexual sphere but who saw marriage as a possibility in the long run, were friendly, had an attractive appearance and had similar interests. Above all, however, they were looking for someone able to accept their disabilities [49].

### 4.3. Level of Tolerance Regarding Risky Life Experiences of Potential Intimate Partners

Our own research found that respondents with disabilities were characterized by significantly more liberal approaches towards almost all risky experiences of potential life partners. Analyses of variance further confirmed that they would tolerate the following past experiences in potential partners significantly more than people without disabilities (statistical significance) would:

- being utterly unfit for the role of parent, including the experience of serving a sentence for using violence against one’s own children and being deprived of parental rights;
- risky sexual experiences: sexually transmitted diseases, providing sex work services, using violence (that includes sexual violence) against previous partners, cheating on previous partners, and having children from previous relationships;
- treatment for a mental illness—schizophrenia or psychosis;
- experiences of addiction to psychoactive substances, including treatment for alcoholism and drug addiction;
- violating legal norms, which resulted, for example, in convictions for violence against a partner and even a stay in a penal institution.

Research on people with disabilities shows that experiences with partner relationships and sexual activity occur less frequently and later in their lives. Feelings of inferiority, common standards of beauty, stigma, and overprotective parenting are cited as reasons. Young adults with disabilities often fail to meet established ideals of beauty. This can make it difficult for them to develop a positive self-image. Therefore, it is often more difficult for them to develop a healthy body image than for people without disabilities. Women in particular are more susceptible to beauty ideals regarding sexuality than men and are judged more harshly in terms of physical beauty and attractiveness. In the case of visible disabilities, rejection from the social environment creates feelings of insecurity and hinders the development of a positive identity. As a result, people with disabilities, especially women with visible disabilities, are less likely to attract partners because social expectations of beauty play an important role when looking for a partner [16,50]. Low self-confidence, feelings of inferiority and lack of attractiveness, overprotectiveness on the part of parents and infantilization of the needs of young adults with disabilities may explain the greater openness of young adults with disabilities to seek potential life partners in a group of people with experiences of risky behavior, as indicated by the results of our own research. Women with disabilities, wanting to maintain their relationships at all costs, become submissive and agree to the partner’s decisions and demands [49]. The results of our own research showed high levels of tolerance of people with disabilities towards the risky life experiences of potential intimate partners and these finding are worth relating to contemporary trends in the dating market. Meta-analyses show that there is an apparent trend indicating persistent, increasing discrimination and stigma experienced in this regard by people growing up with disabilities [22], which justifies the total lack of appropriate selection of life partners by people with disabilities [23,46,51].

It should be emphasized, as C. Friedman’s research shows, that people with disabilities remain isolated and alone in their decisions regarding partner choice, and even professionals do not always help (only 50.8% of institutional staff helped them establish and maintain intimate relationships, 49.9% helped them make favorable intimate relationship choices, while 45.2% supported them in building a satisfying intimate relationship) [8]. Randomly selected partners are likely to become perpetrators of violence in partner relationships in the future and this is associated with public health and social problems, has negative physical and psychological consequences for victims, perpetrators and children [52]. In this context, it is not surprising that, according to a study by C. Friedman, nearly a third of people with disabilities are not satisfied when it comes to their relationships [8].

Immature motives for entering an intimate relationship, high openness to partners, including those burdened by personal problems, and a high level of acceptance of partners’ risky life experiences can translate into impulsive, hasty decisions to start a family. Such attitudes reduce the chances of building a partner relationship that is satisfying for both partners. In the case of people with disabilities, there is a risk of accumulating problems in the relationship, both personal and partnership, which also pose a serious threat to mental health.

### 4.4. Implications

- Support for mental health through the development and formulation of educational programs in the fields of sexual education and education focused on preparation for family life, including content focused on disability and the ability of people with disabilities to realize the role of life partners (sexual, parental) would, in the long term, improve the quality of life of people with disabilities and the social inclusion of people with disabilities.
- People with disabilities need more support to expand their social networks and to become more integrated into society, which would increase their chances of finding a partner outside the group of other people with disabilities. The research revealed that students with disabilities are still more likely to seek life partners among other people with disabilities, thus limiting their own ability to integrate into society.
- People with disabilities, being more likely to be subjected to violence by their life partners (as a result of what is likely to be random, less careful and challenging partner selection), should be particularly supported in the education process towards the formation of assertive attitudes (reinforcing positive perception of oneself, and supportive parents and educators playing crucial roles in the development of young adults with disabilities and their ability to make independent and confident decisions about partnership and sexuality in adulthood).

### 4.5. Strengths and Limitations, Future Studies

Up to now, the subject of research has focused on the opinions of people without disabilities regarding relationships with people with disabilities, or willingness to establish relationships with people with disabilities. 

A strong point of this study is its inclusion of the perspective of people with disabilities.

Among the limitations of individual and independent research, it should be noted that the research was conducted on a group of university students, including students with disabilities. The participation of young adults with disabilities in higher education may be indicative of their positive integration/inclusion experiences to date. Thus, these people are a preferred group among people with disabilities, having adequate cognitive and social resources, such as family support. We could have obtained different findings if the research had also been carried out on groups of young adults with disabilities not in education, working or not working and with experiences of negative social integration. Future research should be expanded to include the opinions of non-students with disabilities and with different educational experiences at earlier stages of education (special, integrated, mainstream schools). In the pages of this article, due to publishing limitations, we refrained from analyzing the gender of the people with disabilities surveyed, and the biological disabilities they declared.

## 5. Conclusions

- Among the motives for entering into permanent intimate relationships, students with disabilities are less likely than students without disabilities to point to mutual love and a partner’s psychological qualities, but are much more likely to indicate improved self-esteem, a partner’s high economic potential, accidental pregnancy and associated social stigma and a shared system of values and interests.
- Students with disabilities, more often than those without disabilities, approve of potential partners who: have physical disabilities, hearing impairments and mild intellectual disability, and are younger. In contrast, they are less likely than students without disabilities to accept partners who care about their physical appearance, are oriented toward a rich cultural and social life; have higher education than them and are older, caring and protective.
- Students with disabilities are characterized by a much more liberal approach towards almost all risky life experiences than students without disabilities. In their potential life partner, they are mainly willing to tolerate experiences indicative of being utterly unfit for the role of parent (including the use of violence against children), risky sexual experiences (including sexually transmitted diseases, the use of sexual violence, committing betrayals of previous partners), experiences of mental health disorders, addiction to psychoactive drugs, and even manifestations of violations of legal norms (including violence against previous partners and terms in penal institutions).

## Figures and Tables

**Table 1:** Motives for starting a family declared by students with and without disabilities.

Motivation for Starting a Family	University Students with Disability Average of Specific Measures	University Students without Disability Average of Specific Measures	*p* Level of Significance of Differences
avoiding “badmouthing” by people due to pregnancy	0.89	0.58	<0.001
proving to others that I can get an attractive woman/man on a permanent basis	1.04	0.73	<0.001
partner’s mental balance	2.33	2.54	=0.001
persuasion by family or friends	0.71	0.51	=0.004
possession of extensive material resources by the partner, for example: apartment, high-end car, money	1.08	0.85	=0.002
similar religious convictions	1.92	1.65	=0.001
proving to myself that I am attractive enough for that kind of man/woman to choose me as a partner	1.3	1.08	=0.005
a feeling of love for their partner	2.80	2.89	=0.022
excellent physical fitness of the partner	1.35	1.15	=0.015
desire to have a family	2.12	2.29	=0.009
partner’s capabilities that guarantee the achievement of high material status in the future (e.g., high education, steady, well-paid work, the chance to inherit property from relatives)	1.45	1.27	=0.016
feeling of being loved by the partner	2.78	2.87	=0.041
past satisfaction regarding sexual intercourse with the partner	1.97	2.07	=0.126
feeling that you need to start a family (e.g., due to age or the fact that others already have families)	1.20	1.09	=0.144
desire to be in a permanent relationship	2.58	2.65	=0.219
similar system of life values	2.41	2.47	=0.241
fear of being a single mother or father	1.31	1.23	=0.361
the partner’s personality appeals to me	2.64	2.68	=0.420
having a constantly available sexual partner	1.40	1.44	=0.557
appearance of the partner that I find attractive	1.82	1.80	=0.789
pressure from the partner to start a family	0.98	0.99	=0.825
the fact of a long-term acquaintance/friendship with a woman or a man	2.09	2.09	=0.979

**Table 2:** Categories of motives for starting a family declared by students with and without disability.

Categories of Motives for Starting a Family	University Students with Disability Average of Synthetic Measures	University Students without Disability Average of Synthetic Measures	*p* Level of Significance of Differences
feeling better about oneself	1.17	0.90	=0.001
mental qualities of the partner	2.48	2.60	=0.010
high economic potential of the partner	1.26	1.06	=0.007
emotional commitment of partners	2.79	2.88	=0.031
pregnancy and the fear of its consequences	1.10	0.91	=0.010
striving to build a community	2.35	2.47	=0.030
a common system of values and interests	2.17	2.06	=0.036
physical qualities of the partner	1.57	1.47	=0.085
pressure (from the environment)	0.85	0.75	=0.145
a sense of internal pressure to start a family	1.65	1.59	=0.220
sexual benefits	1.68	1.75	=0.270

**Table 3:** Qualities of potential life partners accepted by students with and without disabilities.

Categories of Qualities	Accepted Qualities of a Life Partner	University Students with Disability Average of Specific Measures	University Students without Disability Average of Specific Measures	*p* Level of Significance of Differences
disability	physical disability	2.52	2.07	<0.001
	hearing disorders	2.80	2.58	=0.004
	mild intellectual disability	2.16	1.91	=0.005
	visual impairment	2.97	2.87	=0.146
external appearance and care for said appearance	care for external appearance	3.39	3.55	=0.007
	physical attractiveness (as judged by other people)	3.35	3.48	=0.083
	focus on physical fitness	3.53	3.61	=0.110
	being overweight	2.45	2.36	=0.281
	slim figure	3.36	3.35	=0.871
sexual scripts	focus on experimentation in sex life	2.72	2.87	=0.062
	extensive sexual experience (various types of activity/multiple partners)	2.15	2.08	=0.472
	lack of sexual experience (few forms of activity/few partners)	3.10	3.12	=0.749
lifestyle	focus on an active cultural and social life	3.23	3.39	=0.027
	preference for spending leisure time at home, limiting social contacts to the necessary minimum	2.54	2.38	=0.086
	openness to change	3.46	3.51	=0.427
	a career-oriented mindset	2.75	2.79	=0.549
social status	higher education level than mine	3.31	3.48	=0.018
	working in a higher position than mine	3.30	3.42	=0.061
	income higher than mine	3.28	3.34	=0.318
	lower education level than mine	3.21	3.24	=0.704
	working in a lower position than mine	3.27	3.26	=0.894
	income lower than mine	3.16	3.16	=0.955
age	older than me	3.32	3.47	=0.045
	younger than me	3.02	2.83	=0.030
style of functioning in interpersonal relations	thoughtfulness, caring	3.61	3.76	=0.018
	competitive attitude	2.07	1.93	=0.127
	propensity for leadership/dominance	2.19	2.30	=0.213
	independence	3.14	3.21	=0.291
	confidence	3.52	3.57	=0.349
	belief that it is right to share household responsibilities in a partner relationship	3.31	3.34	=0.656
	gentleness, calmness	3.59	3.59	=0.990

**Table 4:** Level of tolerance of risky life experiences of potential life partners declared by students with and without disabilities.

Risky Life Experiences of a Potential Life Partner	University Students with Disability Average of Specific Measures	University Students without Disability Average of Specific Measures	*p* Level of Significance of Differences
has been convicted of violence against children from previous relationships	0.70	0.41	=0.001
has received treatment for alcohol abuse	1.38	1.02	<0.001
has been treated for a mental illness (e.g., schizophrenia, psychosis)	1.49	1.15	=0.001
had a case in court for violence against partner(s)	0.67	0.47	=0.015
has received treatment for drug use	1.23	0.97	=0.010
her/his parental rights to his/her own children have been terminated	0.91	0.70	=0.013
has suffered from sexually transmitted diseases (venereal)	1.38	1.15	=0.016
she/he happened to provide paid sex work service	0.99	0.78	=0.026
forced unacceptable forms of sexual activity on their partner(s)	0.73	0.56	=0.043
was convicted to a term in a penal institution	1.13	0.94	=0.034
happened to cheat on his/her previous partner(s)	1.01	0.83	=0.034
has a child or children from previous relationships	2.38	2.18	=0.042
used e.g., cocaine, amphetamines, ecstasy, LSD, heroin	0.93	0.75	=0.072
she/he happened to use paid sex work services	1.30	1.11	=0.058
abused alcohol (e.g., drinking at least few times a week, sometimes getting drunk, looking for opportunities to drink)	1.08	0.92	=0.072
has used designer drugs	1.26	1.09	=0.060
completely gave up his/her child to other people with regards to the child’s upbringing	1.20	1.08	=0.134
has been treated for mental disorders (e.g., depression)	2.19	2.04	=0.174
has sexual experiences with many partners	2.27	2.16	=0.248
happened to have sold drugs	0.86	0.77	=0.341
has attempted suicide	1.95	1.85	=0.317
lost his/her license for driving under the influence of alcohol	1.30	1.21	=0.331
belonged to a group of hardcore supports who use all means available to destroy the enemies of their sports club	0.85	0.78	=0.457
terminated the pregnancy or consented to the partner’s termination of the pregnancy	1.72	1.63	=0.416
used marijuana or its derivatives	1.82	1.88	=0.589
was/is addicted to using the computer/playing computer games	2.20	2.17	=0.724
was raped	2.54	2.51	=0.823
smoked a lot (more than a pack) of cigarettes a day	1.83	1.85	=0.875
started brawls/arguments after drinking alcohol	1.02	1.02	=0.954
belonged to a neighborhood gang	1.14	1.15	=0.993

**Table 5:** Categories of risky life experiences of potential partners tolerated by students with and without disabilities.

Categories of Tolerated Risky Life Experiences	University Students with Disability Average of Synthetic Measures	University Students without Disability Average of Synthetic Measures	*p* Level of Significance of Differences
being utterly unfit for the role of parent	1.13	0.96	=0.011
risky sexual experiences	1.57	1.41	=0.015
experiences with mental health disorders	1.88	1.68	=0.015
experiences related to breaking legal norms/legal punishment	0.94	0.82	=0.089
experiences related to addiction to psychoactive substances or behavioral addictions	1.41	1.29	=0.099

## Data Availability

The data are owned by University of Rzeszów and are not to be made freely publicly available.

## Appendix A

**Table A1:** Categories of motives highlighted in the “Scale of motives for starting a family”.

Categories of Motives for Starting a Family (Synthetic Measures)	Specific Motives (Question Number in the Tool) (Specific Measures)
physical qualities of the partner	appearance of the partner that I find attractive (q. 2)
	excellent physical fitness of the partner (q. 22)
mental qualities of the partner	the partner’s personality that appeals to me (q. 8)
	partner’s mental balance (q. 17)
emotional commitment of partners	a feeling of love for their partner (q. 3)
	feeling of being loved by the partner (q. 9)
high economic potential of the partner	partner’s capabilities that guarantee the achievement of high material status in the future (e.g., high education, steady, well-paid work, the chance to inherit property from relatives (q. 12)
	possession of extensive material resources by the partner, for example: apartment, high-end car, money (q. 14)
striving to build a community	desire to be in a permanent relationship (q. 1)
	desire to have a family (q. 16)
feeling better about oneself	proving to myself that I am attractive enough for that kind of man/woman to choose me as a partner (q. 13)
	proving to others that I can get an attractive woman/man on a permanent basis (q. 20)
social pressure	pressure from the partner to start a family (q. 5)
	persuasion by family or friends (q. 7)
sexual benefits	having a constantly available sexual partner (q. 15)
	past satisfaction regarding sexual intercourse with the partner (q. 18)
a common system of values and interests	similar system of life values (q. 6)
	similar religious convictions (q. 11)
a sense of internal pressure to form a relationship	the fact of a long-term acquaintance/friendship with a woman or a man (q. 4)
	feeling that you need to start a family (e.g., due to age or the fact that others already have families) (q. 10)
pregnancy and the fear of its consequences	fear of being a single mother or father (q. 19)
	avoiding “badmouthing” by people due to pregnancy (q. 21)

**Table A2:** Qualities of potential life partners included in “Scale of acceptance of qualities of potential life partners”.

Categories of Qualities of a Potential Life Partner (Synthetic Measures)	Specific Qualities (Question Number in the Tool) (Specific Measures)
disability	physical disability (q. 6)
	hearing disorders (q. 4)
	mild intellectual disability (q. 2)
	visual impairment (q. 18)
external appearance and care for said appearance	care for external appearance (q. 8)
	physical attractiveness (as judged by other people) (q. 1)
	focus on physical fitness (q. 25)
	being overweight (q. 27)
	slim figure (q. 21)
sexual scripts	focus on experimentation in sex life (q. 11)
	extensive sexual experience (various types of activity/multiple partners) (q. 3)
	lack of sexual experience (few forms of activity/few partners) (q. 15)
lifestyle	focus on an active cultural and social life (q. 17)
	preference for spending leisure time at home, limiting social contacts to the necessary minimum (q. 14)
	openness to change (q. 22)
	a career-oriented mindset (q. 5)
social status	higher education level than mine (q. 31)
	working in a higher position than mine (q. 13)
	higher income than mine (q. 7)
	lower education level than mine (q. 29)
	working in a lower position than mine (q. 24)
	lower income than mine (q. 9)
age	older than me (q. 26)
	younger than me (q. 16)
style of functioning in interpersonal relations	thoughtfulness, caring (q. 28)
	competitive attitude (q. 19)
	propensity for leadership/dominance (q. 10)
	independence (q. 20)
	confidence (q. 23)
	belief that it is right to share household responsibilities in a partner relationship (q. 30)
	gentleness, calmness (q. 12)

**Table A3:** Risky life experiences of potential life partners included in the “Scale of tolerance level of risky experiences of potential life partners.”.

Categories of Risky Life Experiences (Synthetic Measures)	Risky Life Experiences of a Potential Life Partner (Question Number in the Tool) (Specific Measures)
experiences related to addiction to psychoactive substances or behavioral addictions	has received treatment for alcohol abuse (q. 8)
	has received treatment for drug use (q. 30)
	used e.g., cocaine, amphetamines, ecstasy, LSD, heroin (q. 28)
	abused alcohol (e.g., drinking at least few times a week, sometimes getting drunk, looking for opportunities to drink) (q. 13)
	has used designer drugs (q. 22)
	used marijuana or its derivatives (q. 23)
	was/is addicted to using the computer/playing computer games (q. 1)
	smoked a lot (more than a pack) of cigarettes a day (q. 19)
	started brawls/arguments after drinking alcohol (q. 25)
	lost his/her license for driving under the influence of alcohol (q. 20)
risky sexual experiences	has suffered from sexually transmitted diseases (venereal) (q. 5)
	she/he happened to provide paid sex work service (q. 24)
	forced unacceptable forms of sexual activity on their partner(s) (q. 26)
	happened to cheat on his/her previous partner(s) (q. 29)
	has a child or children from previous relationships (q. 10)
	she/he happened to use paid sex work services (q. 18)
	has sexual experiences with many partners (q. 2)
	was raped (q. 9)
experiences related to breaking legal norms/legal punishment	had a case in court for violence against partner(s) (q. 11)
	was convicted to a term in a penal institution (q. 4)
	happened to have sold drugs (q. 27)
	lost his/her license for driving under the influence of alcohol (q. 20)
	belonged to a group of hardcore supporters who use all means available to destroy the enemies of their sports club (q. 14)
	belonged to a neighborhood gang (q. 16)
	started brawls/arguments after drinking alcohol (q. 25)
	forced unacceptable forms of sexual activity on their partner(s) (q. 26)
	has been convicted of violence against children from previous relationships (q. 3)
experiences with mental health disorders	has been treated for a mental illness (e.g., schizophrenia, psychosis) (q. 7)
	has been treated for mental disorders (e.g., depression) (q. 15)
	has attempted suicide (q. 12)
being utterly unfit for the role of parent	has been convicted of violence against children from previous relationships (q. 3)
	her/his parental rights to his/her own children have been terminated (q. 6)
	completely gave up his/her child to other people with regards to the child’s upbringing (q. 17)
	terminated the pregnancy or consented to the partner’s termination of the pregnancy (q. 21)
